# Osteoprotegerin gene polymorphisms and otosclerosis: an additional genetic association study, multilocus interaction and meta-analysis

**DOI:** 10.1186/s12881-020-01036-8

**Published:** 2020-06-03

**Authors:** Amal Bouzid, Adel Tekari, Fida Jbeli, Amine Chakroun, Kirtal Hansdah, Amal Souissi, Neha Singh, Mohamed Ali Mosrati, Imen Achour, Abdelmonem Ghorbel, Ilhem Charfeddine, Puppala Venkat Ramchander, Saber Masmoudi

**Affiliations:** 1 Laboratory of Molecular and Cellular Screening Processes, Centre of Biotechnology of Sfax, University of Sfax, Road Sidi Mansour Km 6, BP 1177, 3018 Sfax, Tunisia; 2 Department of Otorhinolaryngology, Habib Bourguiba Teaching Hospital, University of Sfax, Avenue El Ferdaws, 3029 Sfax, Tunisia; 3 Institute of Life Sciences, Nalco Square, Chandrasekharpur, Bhubaneswar, Odisha 751023 India

**Keywords:** Otosclerosis, OPG, Polymorphisms, Case-control association, Multilocus association, Meta-analysis

## Abstract

**Background:**

Otosclerosis (OTSC) is among the most common causes of a late-onset hearing loss in adults and is characterized by an abnormal bone growth in the otic capsule. Alteration in the osteoprotegerin (OPG) expression has been suggested in the implication of OTSC pathogenesis.

**Methods:**

A case-control association study of rs2228568, rs7844539, rs3102734 and rs2073618 single nucleotide polymorphisms (SNPs) in the *OPG* gene was performed in a Tunisian-North African population composed of 183 unrelated OTSC patients and 177 healthy subjects. In addition, a multilocus association and a meta-analysis of existing studies were conducted.

**Results:**

Rs3102734 (*p* = 0.013) and rs2073618 (*p* = 0.007) were significantly associated with OTSC, which were predominantly detected in females after multiple corrections. Among the *OPG* studied SNPs, the haplotypes A-A-C-G (*p* = 0.0001) and A-A-C-C (*p* = 0.0004) were significantly associated with OTSC in females. Multilocus association revealed that the SNPs: rs2073618 in *OPG,* rs1800472 in *TGFβ1,* rs39335, rs39350 and rs39374 in *RELN*, and rs494252 in chromosome 11 showed significant OTSC-associated alleles in Tunisian individuals. In addition, meta-analysis of the rs2073618 SNP in Tunisian, Indian and Italian populations revealed evidence of an association with OTSC (OR of 0.826, 95% CI [0.691–0.987], *p* = 0.035).

**Conclusions:**

Our findings suggest that rs3102734 and rs2073618 variants are associated with OTSC in North African ethnic Tunisian population. Meta-analysis of the rs2073618 in three different ethnic population groups indicated an association with OTSC.

## Background

Hearing loss (HL) in humans significantly reduces the quality of life and often leads to social isolation. One of the associated causes of acquired hearing impairment is otosclerosis (OTSC). OTSC is characterized by late-onset progressive sensorineural, conductive or mixed HL. The onset of this disease appears principally in the third decade, while the hot spot age is in the sixth decade [1].

OTSC is a bone-related disorder affecting the otic capsule of the middle ear, which in normal cases undergoes very little remodeling after development and ossification of the tissue. The exact mechanism that controls bone metabolism in the otic capsule and turnover within the auditory structures remains largely unknown. Bone is a dynamic tissue controlled by various biochemical, biomechanical and hormonal stimuli. Bone homeostasis is coordinated at the cellular level by a balance between resorption mediated by the osteoclasts and formation mediated by the osteoblasts [2–4]. An imbalance between both processes occurs under certain pathological conditions that affect the skeleton, which leads to the development of bone diseases [5].

The etiology of OTSC remains complex. It is a multifactorial disease caused by both environmental factors such as a viral infection and genetic factors. Evidence supporting the genetic role includes familial cases and a twin study revealing the high heritability [6]. Earlier studies suggested the autosomal dominant inheritance with reduced penetrance in OTSC [7]. Different populations-based case-control studies have associated a number of single nucleotide polymorphisms (SNPs) in candidate genes with OTSC such as *COL1A1*, *TGFβ1*, *BMP2*, *BMP4*, *AGT*, *ACE* and *FGF2* [8, 9]. In addition, a genome-wide association study identified the region on chr11q13.1 and 7q22.1 in intron 1 to 4 of the *RELN* gene to be associated with OTSC [10]. Recent studies using massive parallel sequencing in OTSC families identified six rare heterozygous *SERPINF1* variants of which three are missense mutations predicted to be deleterious to the protein function [11], and a rare segregating heterozygous frameshift variant in the *MEPE* gene [12].

**Fig. 1:**
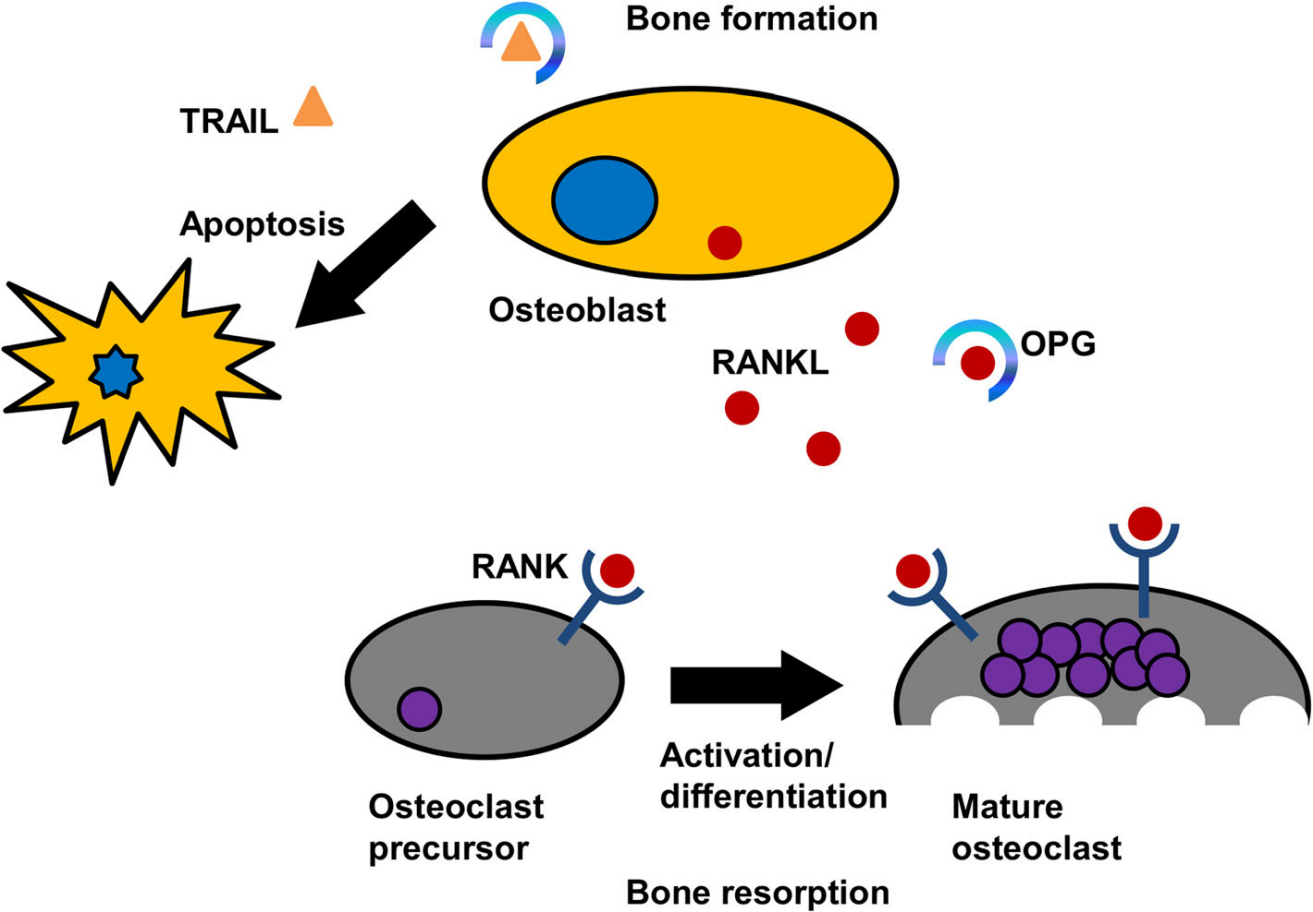
Schematic representation of the cellular and molecular players involved in the RANKL/RANK/OPG signaling axis in bone remodeling. The RANKL from the osteoblast-lineage cells binds to the RANK receptor on osteoclast precursor cells and promotes osteoclasts activation and differentiation, resulting in increased bone resorption. Whereas the OPG decoy receptor binds to RANKL leading to inhibition of osteoclastogenesis. OPG also functions as a decoy receptor for TRAIL, and OPG decreased production mediates the TRAIL-induced apoptosis of osteoblasts leading therefore to bone loss

Animal models and genetically altered mice studies during the past 20 years have greatly increased our knowledge of the factors that regulate the activity and formation of osteoclasts. In particular, the discovery of the receptor activator of nuclear-κB ligand (RANKL)/RANK/osteoprotegerin (OPG) signaling axis has improved our understanding of the role played by osteoblasts in these processes [13]. OPG, also known as osteoclastogenesis-inhibiting factor, is a cytokine receptor and a member of the tumor necrosis factor (TNF) receptor superfamily encoded by the TNF receptor superfamily member 11B (*TNFRSF11B*) gene. The OPG protein is implicated in different signal transduction-interfering biological responses, including apoptosis, cytotoxicity, differentiation and proliferation. It interacts with two common TNF family ligands, TNF-related apoptosis-inducing ligand (TRAIL) and RANKL [14, 15]. The soluble receptor OPG acts as an endogenous decoy receptor towards RANKL (Fig. 1). OPG binds RANKL, thus preventing the interaction of RANKL with, and stimulation of RANK [13, 16]. Therefore, OPG inhibits osteoclast differentiation and maturation and induces the apoptosis of activated osteoclasts as demonstrated in vitro [17] and in vivo [18].

A deficiency in the OPG/RANKL composition induces a range of skeletal diseases such as osteoporosis and bone metastases [19]. Thus, the concentration of RANKL and OPG in bone is a major determinant of bone mass [20]. In addition, OPG prevents the interaction between TRAIL and its death receptors, hence inhibiting TRAIL-induced apoptosis [14].

Although OPG plays an important role in controlling bone turnover, it is considered a relevant candidate for genetic variation in the mechanism of OTSC. For example, OPG knockout mice display abnormal bone remodeling in the otic capsule, similar to human temporal bones with OTSC [21], and overexpression of OPG in transgenic mice causes osteopetrosis [13]. In addition, OPG is expressed at high levels within the inner ear as detected in mice, and is secreted to the perilymph and the surrounding bone, which may serve to inhibit active bone remodeling within the otic capsule [22]. Thus, OPG can be considered to be a potent inhibitory factor of abnormal bone remodeling.

A previous Italian study [23] showed no association between the rs2073618 (N3K) polymorphism in the *OPG* gene and OTSC. However, a recent Indian study revealed *OPG* polymorphisms in OTSC with sex-specific association of rs2073618 in males and rs3102734 in females, whereas no association was resulted for the rs2228568 and rs7844539 *OPG* variants [24]. Therefore, the contribution of *OPG* to OTSC remains controversial. Within this study, we aimed to address these shortcomings by performing a replication association study of the four *OPG* SNPs (rs2228568, rs7844539, rs3102734 and rs2073618) with OTSC by comparing a group of otosclerotic and control Tunisian-North African individuals. In addition, we performed a multilocus association analysis with OTSC of *OPG* rs2073618 SNP and significantly associated SNPs previously reported [25, 26] in the same Tunisian population, located in *TGFβ1, RELN* and chromosome 11. Finally, we performed a comprehensive meta-analysis using the available case and control data from previous studies under different genetic models to further evaluate an association between *OPG* rs2073618 SNP and OTSC.

## Methods

### Patients selection

Patients were recruited by the Otolaryngology Department of the University Hospital of Sfax, Tunisia. The study population comprised 183 unrelated OTSC patients (134 females and 49 males) and 177 unrelated control subjects (99 females and 78 males). The diagnosis of OTSC was based on clinical and audiological investigation and was confirmed during surgery as previously described [25, 26].

### SNPs genotyping assays

Peripheral blood samples (5 mL) were collected from all study subjects in EDTA tubes. Genomic DNA was extracted using a standard phenol-chloroform protocol. Four SNPs (rs2228568, rs7844539, rs3102734 and rs2073618) in the *OPG* gene were selected for a replication study in a Tunisian population based on association data from previous studies [23, 24].

The analysis of SNPs was performed using different allelic discrimination genotyping assays. All polymorphisms of the *OPG* gene were amplified from genomic DNA samples using selected primers (Table 1). For both SNPs, rs7844539 (c.817 + 8A > C) and rs2228568 (c.768A > G), Sanger sequencing was used to detect the variations. For rs3102734 (c.30 + 15C > T), polymerase chain reaction-restriction fragment length polymorphism (PCR-RFLP) method was performed using *HaeIII* restriction enzyme. The rs2073618 (c.9C > G) was genotyped using allele-specific polymerase chain reaction [27].

**Table 1:** Primers sequences used for SNPs genotyping in the *OPG* gene

SNP	Sequence 5′- 3′	PCR-Product (bp)	Genotyping Method
rs3102734	Forward: TGCCGGGACGCTATATATAAC	226	Restiction digestion (*HAEIII)*
	Reverse: TTCTCCCCGCCGGTCCGCT		
rs2073618	Forward 1: CGGGGACCACAATGAACAAC	273	Allele specifc polymerase chain reaction or ASPCR
	Forward 2: CGGGGACCACAATGAACAAG		
	Reverse: CTCTCTCTTGCTGTCTTCCAT		
rs2228568 rs7844539	Forward: TTACAAGGAAACTGGGGAGC	437	Sanger Sequencing
	Reverse: TGTGTGAGGAGGCATTCTTC		

### Association analysis of the OPG

For the four studied SNPs (rs2228568, rs7844539, rs3102734 and rs2073618) in the *OPG* gene, allele and genotype frequencies were calculated using the direct counting method. The Hardy-Weinberg Equilibrium (HWE) for determination of genotype frequency in control groups was examined using the *HWE calculator* as previously described [28]. Statistical power calculation was determined in case-control groups with significance levels set at 5% using a web browser program (http://osse.bii.a-star.edu.sg/). Statistical analysis of association characteristics of the study cohort was performed using SPSS (SPSS version 17.0 for Windows, Chicago, IL). A *p*-value (*p*) of 0.05 was considered significant. Multivariate logistic regression analysis was undertaken to evaluate associations between genotypes and gender of the analyzed *OPG* gene polymorphisms. Statistical evaluation of the level of odds ratio (OR) and 95% confidence intervals (CI) for the allelic models were calculated using the *2BY2* program by Fisher’s exact test [29] to evaluate the risk of *OPG* polymorphisms and OTSC (*p* < 0.05).

### Association analysis in OPG, TGFβ1, RELN and chromosome 11

The multilocus association with OTSC was performed using *SNPAlyze V8 Pro* (Dynacom, Chiba, Japan) for specific polymorphisms reported in the same Tunisian population: rs2073618 (chr8:119964052, C > G) in the *OPG* gene of the present study, rs1800472 (chr19:41847860, C > T) in the *TGFβ1* gene [25], rs39335 (chr7:103453569, A > G), rs39350 (chr7:103467267, C > T) and rs39374 (chr7:103476667, A > G) in the *RELN* gene, and rs494252 (chr11:64600002, G > T) in chromosome 11 [26]. Allelic combination analysis among OTSC case and control groups, were evaluated by the maximum-likelihood method using the expectation-maximization (EM) algorithm. All statistical analyses were two-tailed with the statistical significance level fixed at *p* < 0.05. Permutation *p*-values were calculated by comparing combination frequencies among case and control groups based on 10,000 replications. Only significant combinations that were males, females and both sex-based analysis with a frequency within [0, 10^− 4^] in case or control groups were considered.

Pairwise linkage disequilibrium (LD) analysis of |D’| and r^2^ coefficients was assessed for the six variants (rs2073618, rs1800472, rs39335, rs39350, rs39374, rs494252) using *SNPAlyze V8 Pro* (Dynacom, Chiba, Japan). This analysis was estimated according to HWE model.

### Meta-analysis of rs2073618

A meta-analysis was performed for the rs2073618 (c.9C > G) SNP in the *OPG* gene in order to investigate its genetic effect in previously studied OTSC populations (Tunisian: the current, Indian [24], and Italian [23] populations). The analysis was performed following the fixed-effect model or the random-effects [30] using comprehensive Meta-Analysis Software V2 (Biostat Inc, Englewood, USA). The evaluation of heterogeneity of inter-study variations was performed with Cochran’s Q-test, as a simple Chi-square test [31]. The null hypothesis was that all studies were evaluating the same effect while the rejection of the null hypothesis (*p* < 0.05) would reveal heterogeneity between studies. Egger’s regression and sensitivity analysis were estimated. In addition, an indicator of heterogeneity (I^2^) was evaluated to measure the level of inconsistency across studies [32–35].

## Results

### Association of OPG SNPs with OTSC

**Fig. 2:**
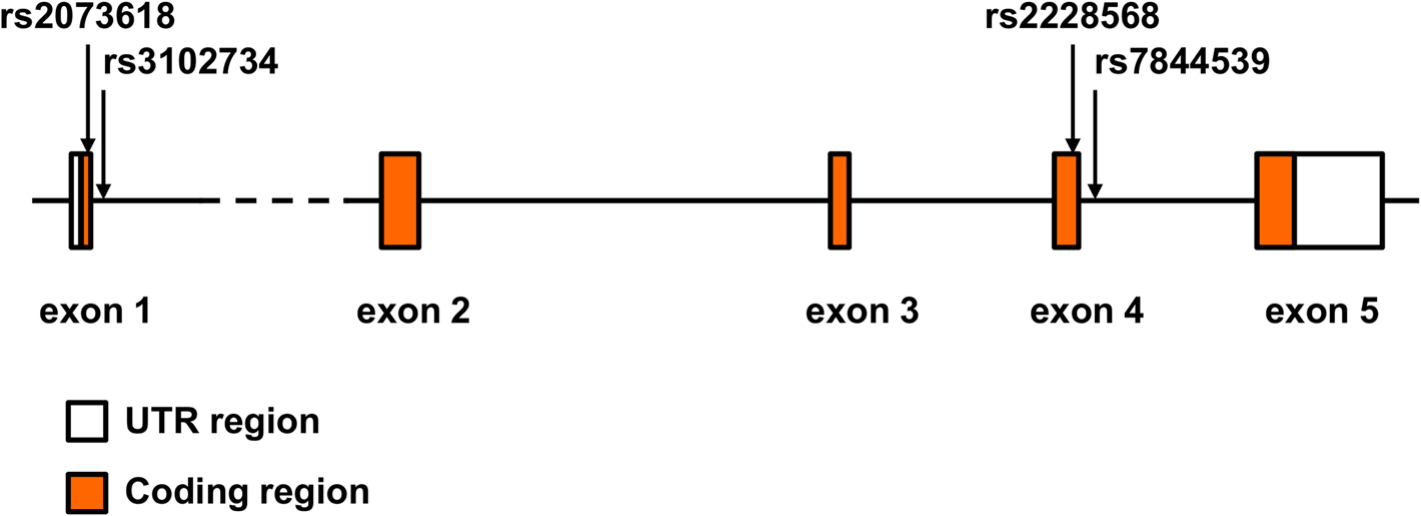
Schematic genomic structure of the *OPG* gene with the positions of the studied polymorphisms. The filled boxes indicate the protein-coding regions and the white boxes indicate the untranslated regions. The variants (rs2228568, rs7844539, rs3102734 and rs2073618) locations within the *OPG* gene are marked with arrows. The sizes of exons/introns and positions of the variants within the *OPG* gene are proportional to the original gene size according to the USC Genome Browser (GRCh37/hg19)

We evaluated the association of previously documented *OPG* SNPs (rs2228568, rs7844539, rs3102734 and rs2073618, Fig. 2) with OTSC in a Tunisian population. HWE analysis revealed no deviations (*p* > 0.001) of the genotype frequencies in the control group for each SNP. Statistical power calculations estimated that at a 5% significance level, the population study had sufficient power to detect SNPs effects with rs3102734 and rs2073618 at 48.5 and 51.1%, respectively, while rs2228568 and rs7844539 had limited power to detect an effect at a significance level of 4.7%.

Case-control association analyses revealed no statistically significant genetic differences between patients with OTSC and controls for the polymorphisms rs2228568 (*p* = 0.483) and rs7844539 (*p* = 0.483) within exon 4 of the *OPG* gene. Statistically significant associations were detected between the rs3102734 (*p* = 0.013) and rs2073618 (*p* = 0.007), located within exon 1 of the *OPG* gene, and patients with OTSC compared to healthy controls. Significant allelic associations between the rs3102734 and rs2073618 variants, and OTSC were detected with *p* = 0.014 and *p* = 0.0041, respectively (Table 2). The minor allele frequencies (MAFs) in the patients with OTSC accounted for 0.041 in rs3102734, and 0.71 in rs2073618, while the respective MAFs in the healthy controls was 0.09 and 0.619, respectively. In addition, a female gender association with OTSC was detected in rs3102734 and rs2073618 variants (*p* = 0.046, *p* = 0.002) but not in males (*p* = 0.096, *p* = 0.416), whereas no gender effect was obtained for both rs2228568 and rs7844539 SNPs (Table 3).

**Table 2:** Genotype and allele frequencies of the analyzed polymorphisms of the *OPG* gene for OTSC and healthy control subjects

SNP ID	Genotype Frequency (%)			X^2^-Test *p* < 0.05	OR (95% CI)	Allele Frequency (%)			*p*-value
		Cases	Controls				Cases	Controls	
rs2228568 (c.768A > G)	A/A	78.6	76	0.483					0.90
	A/G	18.1	22.4			A	87.6	87.2	
	G/G	3.3	1.6			G	12.4	12.8	
rs7844539 (c.817 + 8A > C)	A/A	78.6	76	0.483					0.90
	A/C	18.1	22.4			A	87.6	87.2	
	C/C	3.3	1.6			C	12.4	12.8	
rs3102734 (c.30 + 15C > T)	C/C	92.9	84.2	**0.013**	Reference				**0.014**
	C/T	6	14.1		0.379 (0.04–3.613)	C	95.9	91.2	
	T/T	1.1	1.7		0.287 (0.116–0.708)	T	4.1	8.8	
rs2073618 (c.9C > G)	C/C	14.8	19.9	**0.007**	Reference				**0.0041**
	C/G	30.1	40.4		0.494 (0.235–1.037)	C	29.8	40.1	
	G/G	55.1	39.8		0.482 (0.265–0.876)	G	70.2	59.9	

*SNP* Single-nucleotide polymorphism; *OR* Odds ratio; *CI* Confidence interval. Statistical significance were not obtained with otosclerotic and control samples in the rs2228568 and rs7844539 polymorphisms, while significant values were detected for the rs3102734 and rs2073618 polymorphisms (in bold). Besides, both SNPs revealed an allelic association with OTSC

**Table 3:** Association between genotype and gender of the analyzed polymorphisms of the *OPG* gene

SNP ID	*p*-value (Females)	*p*-value (Males)
rs2228568 (c.768A > G)	0.442	0.467
rs7844539 (c.817 + 8A > C)	0.442	0.467
rs3102734 (c.30 + 15C > T)	**0.046**	0.096
rs2073618 (c.9C > G)	**0.002**	0.416

*SNP* single-nucleotide polymorphism. Significant associations between the polymorphisms rs3102734 and rs2073618 and gender were obtained in females only, which are indicated in bold

### OPG haplotypes association with OTSC and linkage disequilibrium analysis

In order to evaluate the potential effects of allelic combinations on the risk of OTSC, haplotype analysis was performed for the four described *OPG* SNPs [rs2228568 (A > G), rs7844539 (A > C), rs3102734 (C > T), and rs2073618 (C > G)] in the Tunisian population. Five common haplotypes resulted within the *OPG* gene in OTSC cases and controls, with two being significant (A-A-C-G, *p* = 0.0135 and A-A-C-C, *p* = 0.0209, Table 4). Similar results to the genotype analysis, sex-stratified haplotypes analysis revealed gender-specific association of female groups with OTSC for the rs2073618 (c.9C > G). The haplotype A-A-C-G with “G” allele was significantly increased (*p* = 0.0001) in cases suggested an increased risk of OTSC in females while the A-A-C-C with “C” allele was significantly increased (*p* = 0.0004) in controls suggested a reduced risk of OTSC in females. The analysis of the haplotype A-A-C-G revealed that the disease-associated “G” allele in the Tunisian population is in line with the previously reported Indian study but with male-specific association.

**Table 4:** *OPG* haplotype structure and frequencies

	Haplotype frequency								
	Overall			Females			Males		
Haplotype	Cases	Controls	*p*-value	Cases	Controls	*p*-value	Cases	Controls	*p*-value
A-A-C-G	62.19	48.59	**0.0135**	63.83	37.06	**0.0001**	57.65	68.42	0.2658
A-A-C-C	22.28	33.5	**0.0209**	21.32	42.54	**0.0004**	24.89	18.42	0.4373
A-A-T-G	4.39	6.42	0.4015	4.84	8.36	0.2638	29.8	5.26	0.5453
C-G-C-C	5.76	3.2	0.2958	5.49	3.76	0.5611	6.69	2.63	0.3632
C-G-T-G	0.69	1.19	0.6137	0.62	1.83	0.3465	0.97	0	0.5439

Four SNPs in the following order: rs2228568, rs7844539, rs3102734, rs2073618, were used to analyze the haplotypes within the *OPG* gene in cases and controls. P-values are based on 10,000 permutations. Two significant haplotypes were detected: A-A-C-G and A-A-C-C (values are indicated in bold)

### Multilocus association with OTSC

Allele combinations were examined in order to evaluate the multilocus association effect of selected SNPs with the risk of OTSC. Therefore, the rs2073618 (C > G) SNP in the *OPG* gene was considered, and previously reported SNPs, located in three different regions rs1800472 (C > T) in the *TGFβ1* gene [25], rs39335 (A > G), rs39350 (C > T) and rs39374 (A > G) in the *RELN* gene and rs494252 (G > T) in the chromosome 11 [26]. Statistical analysis was carried out for the six SNPs (Table 5) and resulted in four common allele combinations detected in otosclerotic and control patients (Table 6). Among these, two allele combinations resulted in significant differences between OTSC cases and controls with a sex-specific association: C-C-A-C-A-G (*p* = 0.0008**)** for females and C-C-A-T-G-G (*p* = 0.0094**)** for males.

**Table 5:** Statistical evaluation of six selected SNPs

	Number of genotypes	Number of alleles	Exact *p*-value	FDR *q*-value
rs2073618	3	2	2.6037 E-2	9.55 E-2
rs1800472	2	2	1	6.29 E-1
rs39335	3	2	7.4535 E-2	1.06 E-1
rs39350	3	2	3.1866 E-1	3.61 E-1
rs39374	3	2	4.2345 E-1	4.31 E-1
rs494252	2	2	1	5.94 E-1

*FDR* False discovery rate. Six SNPs in the following order, rs2073618, rs1800472, rs39335, rs39350, rs39374 and rs494252 were considered to analyze association with OTSC

**Table 6:** Frequencies of common allele combinations of *OPG, TGFβ1, RELN* and *Chromosome 11* SNPs in OTSC cases and controls

	Frequency								
	Over all			Females			Males		
Allele combinations	Cases	Controls	*p*-value	Cases	Controls	*p*-value	Cases	Controls	*p*-value
G-C-A-C-A-G	0.2703	0.2322	0.4113	0.2715	0.2367	0.5416	0.1387	0.3055	**0.0349**
G-C-G-C-A-G	0.1923	0.1662	0.5235	0.1888	0.1735	0.7609	0.2541	0.1662	0.2594
C-C-A-C-A-G	0.0669	0.1869	**0.0004**	0.0476	0.1732	**0.0008**	0.2363	0.1019	0.0609
C-C-A-T-G-G	0.1215	0.0533	**0.0281**	0.1077	0.0779	0.4392	0.0179	0.1569	**0.0094**

The allele combination analysis of the six SNPs in the following order: rs2073618, rs1800472, rs39335, rs39350, rs39374 and rs494252 revealed significant differences between cases and controls with sex-specific associations in C-C-A-C-A-G for females and G-C-A-C-A-G and C-C-A-T-G-G for males

Furthermore, pairwise linkage disequilibrium was calculated according to D’ and r^2^ statistics for all possible two-way comparisons between the six following SNPs: rs2073618 (C > G) in the *OPG* gene, rs1800472 (C > T) in the *TGFβ1* gene*,* rs39335 (A > G), rs39350 (C > T) and rs39374 (A > G) in the *RELN* gene and rs494252 (G > T) in chromosome 11. The degree of LD between these SNPs resulted in strong linkage disequilibrium (D’ > 0.8) for different two-way combinations. The rs1800472 SNP was in complete LD with the three SNPs (rs2073618, rs39350 and rs39374), but also the rs494252 was in complete LD with rs39350 and rs39374 SNPs (Table 7).

**Table 7:** Linkage disequilibrium between significant associated SNPs in *OPG, TGFβ1, RELN* and Chromosome 11 with OTSC

	rs2073618	rs1800472	rs39335	rs39350	rs39374	rs494252
rs2073618						
rs1800472	**1**					
rs39335	−0.4537	0.3508				
rs39350	0.2804	**−1**	**−0.8309**			
rs39374	0.1915	**−1**	−**0.9229**	**0.8643**		
rs494252	−0.0263	0.2859	−0.7712	**− 1**	**− 1**	

Results in bold indicate D’ > 0.8: SNPs in strong linkage disequilibrium

### Meta-analysis of the association of rs2073618 SNP in the OPG gene with risk of OTSC

**Fig. 3:**
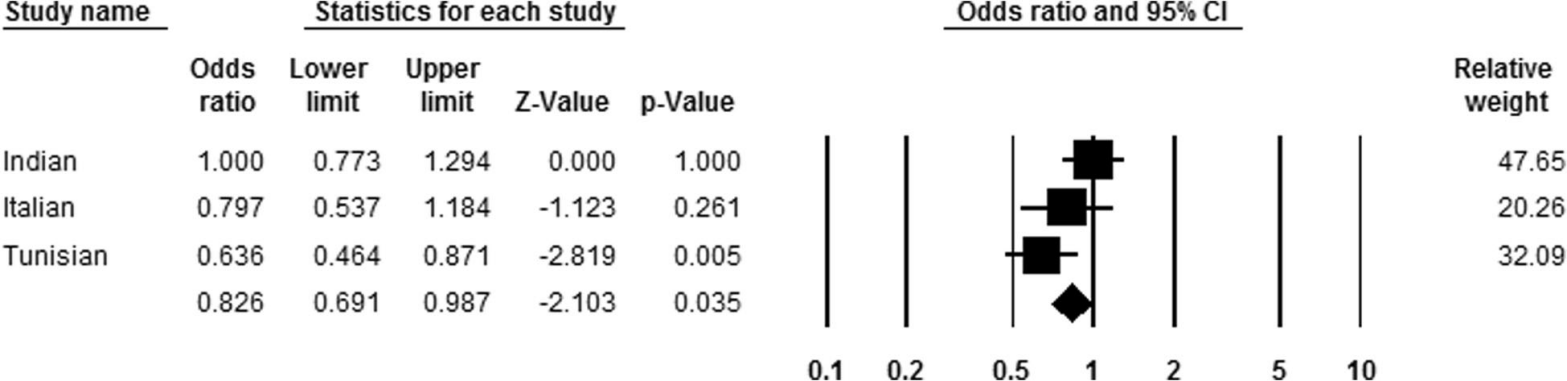
Forest plot of Indian, Italian and Tunisian (current) studies for rs2073618 in the *OPG* gene. An association was detected between rs2073618 SNP and OTSC

A meta-analysis of the rs2073618 (c.9C > G) SNP in the *OPG* gene was conducted in Tunisian (the present study), Indian and Italian populations which included a total of 528 OTSC and 511 control samples. Statistical significance was evaluated through Z and *p*-value. The resulted association outcomes were in line with the previously reported in Indian population [24]. A random effects model was selected with a significant heterogeneity. The forest plot revealed a correlation of the rs2073618 SNP with OTSC, yielded a significant summary (OR of 0.826, 95% CI [0.691–0.987], *p* = 0.035, Fig. 3). In addition, the combined results suggested a robust and significant association of the rs2073618 (c.9C > G) with OTSC under different genetic models: dominant model *p* = 0.006, OR = 0.701, 95% CI [0.545–0.901], recessive model *p* = 0.076, OR  = 0.737, 95% CI [0.527–1.032], heterozygous model *p* = 0.022, OR = 0.729, 95% CI (0.557–0.955), and homozygous model *p* = 0.02, OR  = 0.647, 95% CI [0.449–0.933].

**Fig. 4:**
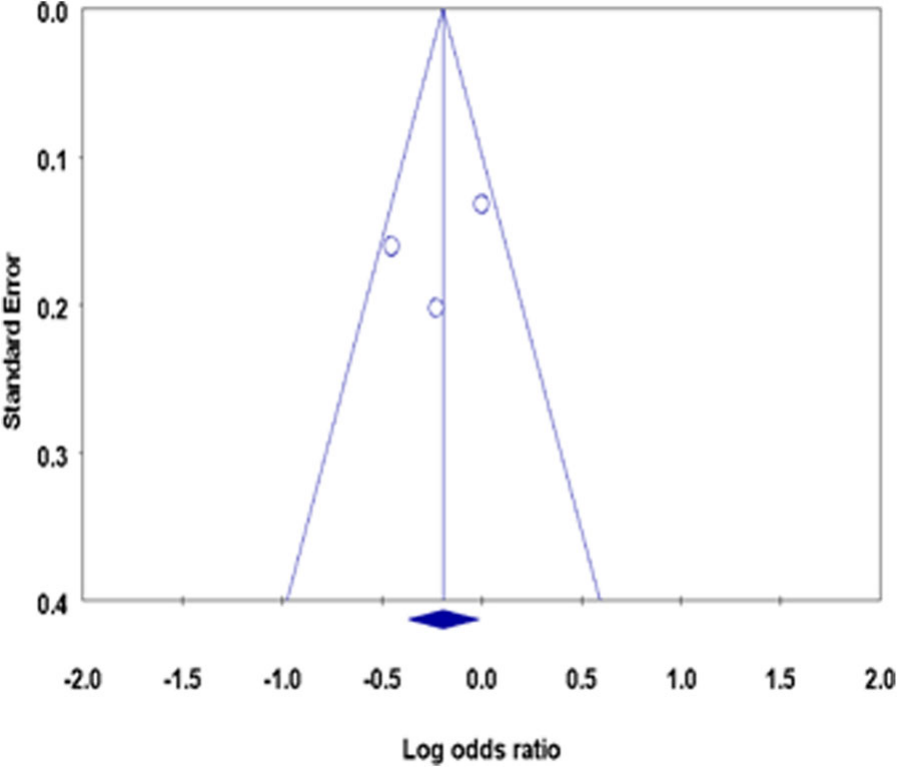
Begg’s funnel plot of standard error by Log odds ratio of the rs2073618 SNP in the *OPG* gene. No publication bias was obtained between the different studies (Indian, Italian and Tunisian)

Finally, sensitivity analysis was performed to reveal the influence of each individual study on the overall meta-analysis correlation. No single study affected the combined OR significantly, suggesting that this meta-analysis is relatively stable. Begg’s funnel plot (Fig. 4) and Egger regression test (*p* = 0.63) showed no publication bias within the studies included in the meta-analysis.

## Discussion

Metabolic bone diseases and injuries of bones are major causes of human skeletal malformations resulting in abnormal mineralized tissue microarchitecture. These are serious health concerns with a severe socio-economic impact [36–38]. Amongst others, OTSC affects several million individuals worldwide with a late-onset of HL and represents a major problem that deserves greater attention. In recent years, a lot of effort has been made to identify the disease-causing genes of OTSC, resulting in the determination of ten loci [39, 40]. Mapping these monogenic loci has not resulted in the recognition of any OTSC causative gene to date. Only a genome-wide analysis study identified the genetic association of the *RELN* gene with OTSC [10].

OPG is a glycoprotein which inhibits osteoclast formation, maturation, osteolysis and induces the apoptosis of activated cells. The main function of OPG is to regulate the normal bone turnover with a balanced bone resorption and formation. OPG is secreted by osteoblasts and mesenchymal stem/stromal cells in order to protect the skeleton from excessive bone resorption by binding to RANKL and preventing it from interacting with RANK, the osteoclastic cell surface receptor [41]. Alteration in the *OPG* gene expression has been suggested to be involved in OTSC. For instance, research in animals genetically unable to produce OPG, revealed HL and histopathology of the temporal bone consistent with that observed in OTSC [21]. Karosi et al. [42] and Priyadarshi et al. [24] reported a reduced or missing *OPG* gene expression in the stapes tissues obtained from otosclerotic patients, however, the exact mechanism by which *OPG* gene expression is altered in OTSC patients is not fully understood.

Within this study, we aimed to evaluate the association of *OPG* gene single nucleotide polymorphisms and OTSC. For that purpose, we performed a replication association study by screening for *OPG* variants in a North African Tunisian subpopulation affected with OTSC compared to a control group of healthy patients. The association results of two SNPs (rs2228568 and rs7844539) of the *OPG* gene in Tunisian OTSC patients confirmed previous observations within the Indian population which found no significant association with OTSC.

Priyadarshi et al. [24] reported a sex-specific association between OTSC and *OPG* gene polymorphisms c.9C > G (rs2073618) and c.30 + 15C > T (rs3102734) in Indian population. Our data confirm the rs2073618 and rs3102734 SNPs sex-specific association with OTSC in the Tunisian population.

The rs2073618 was the only SNP genotyped in Indian and Italian populations. A meta-analysis based on the genotype and allele frequency distribution was performed in this study and further provided evidence of a correlation of the rs2073618 SNP and OTSC suggesting that OPG may have an important role in the pathogenesis of OTSC.

Population-based association studies have identified a number of genes associated with OTSC. These studies have included British, Belgian, Tunisian, and Indian populations. The genes that have been identified include members of the transforming growth factor beta (TGFβ) superfamily, namely TGFβ1, and bone morphogenetic proteins (BMP2 and BMP4). Further evidence for the role of the TGFβ superfamily in the progression of OTSC has been described in several protein expression studies [25, 43–45], showing the presence of the TGFβ superfamily in active otosclerotic foci. In addition, variants in the *RELN* gene appear to be associated with OTSC in a number of studies [8, 26, 46, 47] including British, Italian, Belgian-Dutch and Tunisian populations. Within this study, a multilocus association of six SNPs, including rs2073618 in *OPG*, rs1800472 in *TGFβ1*, rs39335, rs39350 and rs39374 in *RELN* and rs494252 in chromosome 11, revealed significant allele combinations with sex-specific association in OTSC subjects. A previous study assessed the relationship between *OPG* variants and bone mineral density (BMD) or osteoporotic fractures in postmenopausal Chinese. The study showed a significant association between rs2073618 and both BMD and osteoporotic fractures [48]. In addition, the same SNP in the *OPG* gene appears to be associated with a decreased BMD in a case-control study performed in Mexican-Mestizo women with rheumatoid arthritis [49]. Taken together, alteration in the *OPG* gene is related to abnormal bone metabolism and a number of skeletal pathologies including not only OTSC but also age-related bone diseases such as osteoporosis and rheumatoid arthritis.

To the best of our knowledge, this is the first study that shows an association between a SNP in the *OPG* gene and OTSC in a North African population, and supports previous reports of OTSC in an Indian population. Further investigations include functional validation of the rs2073618 SNP through in vivo insertion of the *OPG* promoter and monitor whether it affects DNA-protein complex formation and promoter activity. In addition, with regard to the importance and synergy of the RANKL/RANK/OPG in bone turnover, the remaining genes should be investigated in OTSC case-control association studies.

## Conclusions

In conclusion, we evaluated the association between the *OPG* SNPs and OTSC and found that the rs3102734 and rs2073618 SNPs are linked to the onset of OTSC. This further supports the hypothesis that OPG may play an important role in bone turnover and metabolism within the otic capsule, and those functional polymorphisms within this gene may lead to the development of bone malformations such as OTSC.

## Data Availability

The data that support the findings of this study are not publicly available. Data are however available from the corresponding author upon reasonable request.

## Abbreviations

ACE: Angiotensin-converting enzyme
AGT: Angiotensinogen
BMD: Bone mineral density
BMP2: Bone morphogenetic protein 2
BMP4: Bone morphogenetic protein 4
CI: Confidence interval
COL1A1: Collagen type I, alpha 1
EDTA: Ethylenediaminetetraacetic acid
EM: Expectation–maximization
FGF2: Fibroblast growth factor 2
HL: Hearing loss
HWE: Hardy-Weinberg equilibrium
LD: Linkage disequilibrium
MEPE: Matrix extracellular phosphoglycoprotein
OPG: Osteoprotegerin
OR: Odds ratio
OTSC: Otosclerosis
PCR-RFLP: Polymerase chain reaction-restriction fragment length polymorphism
RANK: Receptor activator of nuclear kappa-B
RANKL: Receptor activator of nuclear kappa-B ligand
RELN: Reelin
SERPINF1: Serpin family F member 1
SNP: Single nucleotide polymorphism
TGFβ1: Transforming growth factor beta 1
TNF: Tumor necrosis factor
TRAIL: Tumor necrosis factor related apoptosis inducing ligand
